# Comparative transcriptomic profile analysis of fed-batch cultures expressing different recombinant proteins in *Escherichia coli*

**DOI:** 10.1186/2191-0855-1-33

**Published:** 2011-10-22

**Authors:** Ashish K Sharma, Shubhashree Mahalik, Chaitali Ghosh, Anuradha B Singh, Krishna J Mukherjee

**Affiliations:** 1 School of Biotechnology, Jawaharlal Nehru University, New delhi-67, India

**Keywords:** Transcriptomic profiling, recombinant, fed-batch, *Escherichia coli*

## Abstract

There is a need to elucidate the product specific features of the metabolic stress response of the host cell to the induction of recombinant protein synthesis. For this, the method of choice is transcriptomic profiling which provides a better insight into the changes taking place in complex global metabolic networks. The transcriptomic profiles of three fed-batch cultures expressing different proteins viz. recombinant human interferon-beta (rhIFN-β), Xylanase and Green Fluorescence Protein (GFP) were compared post induction. We observed a depression in the nutrient uptake and utilization pathways, which was common for all the three expressed proteins. Thus glycerol transporters and genes involved in ATP synthesis as well as aerobic respiration were severely down-regulated. On the other hand the amino acid uptake and biosynthesis genes were significantly repressed only when soluble proteins were expressed under different promoters, but not when the product was expressed as an inclusion body (IB). High level expression under the T7 promoter (rhIFN-β and xylanase) triggered the cellular degradation machinery like the osmoprotectants, proteases and mRNA degradation genes which were highly up-regulated, while this trend was not true with GFP expression under the comparatively weaker *ara *promoter. The design of a better host platform for recombinant protein production thus needs to take into account the specific nature of the cellular response to protein expression.

## Introduction

The wide variability in the expression levels of recombinant proteins in *Escherichia coli *remains a major challenge for biotechnologists. While some proteins are routinely expressed at 30-40% of total cellular protein (TCP) (Joly and Swartz 1997; Kim et al. 2003; Suzuki et al. 2006), others may reach a maximum of only 5% of TCP (Kiefer et al. 2000). The uses of strong promoters, removal of codon bias and media design are favored strategies for improving recombinant protein yield (Acosta-Rivero et al. 2002; Hale and Thompson 1998). It is important to note that most scale up strategies involving high cell density cultures tend to increase biomass concentrations and hence volumetric product concentrations rather than the specific product yield in terms of product formed per unit biomass (Y_p/x_). This yield remains an intrinsic property of the host-vector-gene combination used for expression. Improvements in host vector systems has tended to focus on developing high copy number plasmids with strong tightly regulatable promoters (Bowers et al. 2004; Jones et al. 2000; Wild and Szybalski 2004) along with protease free and recombination deficient strains (Meerman and Georgiou 1994; Ratelade et al. 2009). The focus has thus primarily been on enhancing the metabolic flux of the recombinant protein expression pathway, with few studies on analyzing how the gene products interact with the host cell machinery to depress its own expression.

It has been routinely observed that the specific growth rate of recombinant cultures declines post induction. Earlier authors had correlated this decline to be a measure of the metabolic burden associated with recombinant production (Bentley et al. 1990; Seo and Bailey 1985). It was postulated that the availability of critical metabolites was reduced since they were diverted to product formation, leading to a concomitant decline in the specific growth rate (Babaeipour et al. 2007). It is therefore to be expected that the decline in growth should be most severe when expression levels are maximum. However in most cases there seems to be no such correlation since severe growth retardation is observed when some proteins are expressed in fairly low amounts (Bhattacharya et al. 2005) whereas high level expression of other proteins cause little or no growth retardation (Srivastava and Mukherjee 2005; Vaiphei et al. 2009). The metabolic burden hypothesis is also unable to explain the large variability observed in the levels of recombinant protein yield.

Recent studies on the transcriptomic profiling of recombinant cultures has improved our understanding on the nature of cellular stress associated with over-expression of recombinant proteins (Haddadin and Harcum 2005). Global regulators are triggered in response to induction and these in turn up/down-regulate sets of genes involved in a range of cellular functions (Perez-Rueda and Collado-Vides 2000; Perrenoud and Sauer 2005). These include genes for central carbon metabolism glycolysis, Entner-Doudoroff pathway, pentose phosphate pathway (PPP), tricarboxylic acid (TCA) pathway, glyoxylate shunt (GS), respiration, transport, anabolism, catabolism and macromolecular degradation, protein biosynthesis, cell division, stress response, flagellar and chemotaxis system. This coordinated response of the host mimics many features of the heat shock, osmotic shock, oxidative stress and stringent responses (Gill et al. 2000; Kurland and Dong 1996). This results in the decline of both growth and product formation rates. Thus transcriptomic data reveals a more complex picture of the host response where the cell dynamically reacts to the stress associated with recombinant protein expression. In this work we have tried to extend this analysis by two ways. Firstly we have mimicked industrial scale fermentation where complex media is used to obtain a combination of high cell densities along with high specific growth rates. The latter allows high specific product formation rates and thus product yields are significantly higher in complex media. The transcriptomic profiling of such cultures could provide a more meaningful picture of the cellular physiology under conditions of hyper-expression. We have also attempted to overcome the problems of monitoring cultures grown in complex media by online measurement of metabolic activity like OUR, CER, etc. Secondly we have looked at the variability in cellular stress responses as a function of the nature of the expressed protein. For this we choose three proteins viz. rhIFN-β, Xylanase and GFP, where the bioprocess parameters for high level expression has been previously optimized in our lab. A primary reason for choosing these three proteins was to analyse the difference in the transcriptomic profile when two soluble proteins were expressed under different expression systems and also to see the variability in the cellular response when expression is in the form of inclusion bodies (rhIFN-β) or as a soluble protein (xylanase). In all these cases there is a large diversion of the metabolic flux towards recombinant protein synthesis and thus according to the 'metabolic burden' hypothesis the cellular stress response should be similar. However we observed significant difference in the up/down regulation of genes demonstrating that the cellular response is a function of the gene product and the expression system used.

## Materials and methods

### Chemicals and reagents

Media and bulk chemicals were purchased from local manufacturers, Himedia, Qualigens, and Merck. Media used were LB (Luria-Bertani media containing yeast extract 5 g, tryptone 10 g, and NaCl 10 g/L, pH 7.2), TB (Terrifc broth containing yeast extract 24 g, tryptone 12 g/L, and 0.4% glycerol, pH 7.2). IPTG (1 mM), ampicillin and chloramphenicol were from Sigma, USA. Restriction and modifying enzymes were purchased from MBI Fermentas. All other chemicals were of analytical grade and obtained from local manufacturers.

### Strains and plasmids

*Escherichia coli *strain BL-21 (DE3) [(F^- ^*ompT hsdSB*(*rB*^-^*mB*^-^) *recA1 gal dcm _*(*DE3*) (*lacI lac UV5-T7 gene 1ind1 Sam7 nin5*)] was obtained from Novagen, USA. Strain DH5α (*supE44*_*lac*U169 (_*80 lacZ *_*M15*) *hsdR17 recA1 endA1 gyrA96 thi-1 relA1*) was obtained from Amersham Biosciences, USA. Plasmid pET22b (Amp^R^) was from Novagen, USA, pRSET B (Amp^R^) from Invitrogen, Netherland and pBAD33 (Chloramphenicol^R^) from J. Beckwith, USA.

### Cloning & expression of Representative proteins

rhIFN-β gene was inserted downstream of the T7 promoter in a pET22b expression vector and transformed into *E.coli *BL-21(DE3) cells. rhIFN-β gene was synthesized using SOEing PCR where all the non optimal codons were replaced with optimal codons.

The complete xylanase gene fragment was amplified using M13 forward and XylR primers and a hexahistidine fused xylanase was cloned into the pRSET B vector. This construct was named pRSX and showed soluble cytoplasmic expression.

Cloning of GFP gene into pBAD33 was done by digesting pET14b-GFP (obtained from ICGEB, India) with enzymes *Xba*I and *Hind*III and ligating it into plasmid pBAD33 (which does not contain any ribosome binding site). GFP was cloned under the *ara *promoter which is a tightly regulated promoter.

### High cell density cultivation

A freshly transformed single colony of each clone was inoculated in 10 ml Terrific Broth (TB) containing 100 μg/ml (1×) ampicillin and grown over night. This culture was used to inoculate 200 ml TB having the same antibiotic concentration and grown further for 8 h (OD~ 7). This was used as an inoculum for the fermenter (Sartorius Biostat B Plus) containing TB medium & 1× antibiotic. Temperature, pH and initial Dissolved Oxygen (DO) were set at 37°C, 7.0 and 100% respectively with the initial stirrer at 250 rpm. DO was cascaded with stirrer and maintained at 40%. The airflow rate was kept at 2 l/m. The medium pH was set at 7.0 and controlled by automatic addition of 1 N HCl or NaOH. Sigma Antifoam 289 was added when required. The feeding solution which comprises 12% peptone, 12% Yeast Extract and 18% Glycerol was fed so as to maintain the pre-induction μ at 0.3 h^-1^. The culture was initially grown in a batch mode till 10-12 OD and then the feed was attached. In order to support the growth at a constant specific growth rate of 0.3 h^-1^, the feed rate was increased exponentially using the equation F = F_o_e^μt^, where F_o _is the initial flow rate, F is the flow rate at any given time, μ is the specific growth rate and t is time in hours. Simultaneously, the metabolic activity of the cultures was estimated indirectly by observing the Oxygen Uptake Rate (OUR) and Carbon Emission Rate (CER) which was measured by an exit gas analyser (FerMac 368, Electrolab Ltd, Tewkesbury, UK). RPM is also a useful online indicator of the oxygen transfer rate which matches the oxygen uptake rate (OUR) when dissolved oxygen is at steady state. Since throughout the experiment, dissolved oxygen was maintained at 40% by cascading RPM with dissolved oxygen, we could correlate these parameters with the metabolic activity of the culture (Gupta et al. 1999). Thus a plot of OUR versus RPM^2^, gave a straight line (Additional File 1) and this provided us with a cross check on the measured values of OUR. This was used to estimate the online metabolic activity of the culture post induction which allowed us to design the post induction feeding strategy without allowing substrate buildup in the media. From the pH profile it was ensured that there was no acetate accumulation and both acetate and glycerol levels were monitored using the Megazyme Acetic Acid kit (KACETRM; Megazyme International Ireland Limited) and using the Megazyme Glycerol kit (K-GCROL; Megazyme International Ireland Limited) respectively, to confirm that there was no overflow metabolism.

### Transcriptomic Profiling

Samples from fed batch fermentations of rhIFN-β, Xylanase and GFP were collected at four time points (0 h, 2 h, 4 h, and 6 h) after induction. 0 h (uninduced) samples were taken as a control for every run. The cDNA synthesis, labelling (biotin) and hybridization (Affymetrix GeneChip *E.coli *genome 2.0 array) were performed according to the Affymetrix GeneChip expression analysis protocols. Washing, staining and amplification were carried out in an Affymetrix GeneChip^® ^Fluidics Station 450. Affymetrix GeneChip^® ^scanner 3000 was used to scan the microarrays. Quantification and acquisition of array images were done using Affymetrix Gene Chip Operating Software (GCOS) version 1.4. Three types of detection call (i.e., present, absent, or marginal) were calculated using statistical expression algorithm and average normalization was performed. Hybridization and spike controls were used.

Subsequent data analysis was performed using GeneSpring GX11.5 software (Agilent Technologies, USA). RMA algorithm was used for data summarization (Bolstad et al. 2003) and quality control of samples was assessed by principle component analysis (PCA). Fold change was calculated as time point/uninduced control (0 h). Normalized signal intensities of each gene on chips were converted to log2 values, and compared between experiments.

The microarray data series of fed batch runs have been deposited to the Gene Expression Omnibus database at NCBI under the accession number GSE28412 for rhIFN-β (GEO; http://www.ncbi.nlm.nih.gov/geo/query/acc.cgi?acc=GSE28412), GSE29439 for xylanase (GEO; http://www.ncbi.nlm.nih.gov/geo/query/acc.cgi?acc=GSE29439) and GSE29440 for GFP (GEO; http://www.ncbi.nlm.nih.gov/geo/query/acc.cgi?acc=GSE29440).

### Experimental design for data analysis

The data set was filtered and genes with ≥ 2 fold change were selected for further analysis. The comparison was done across all time points for all 3 sets of recombinant protein and the common set of up/down-regulated gene were used for further analysis. The comparison set is shown as a Venn diagram in Additional file 2a.

To analyze the similarities in the response to rhIFN-β, Xylanase and GFP production, common genes in all the three gene sets were extracted and shown in Additional file 2b, e and Additional file 3.

Next, to analyse the effect of hyper-expression of recombinant protein under a strong promoter, the list of genes that were exclusively up/down-regulated in the time course profiles of rhIFN-β and Xylanase but not in GFP were extracted from the Venn diagram as shown in Additional file 2.c, f and Additional file 4.

**Figure 1:**
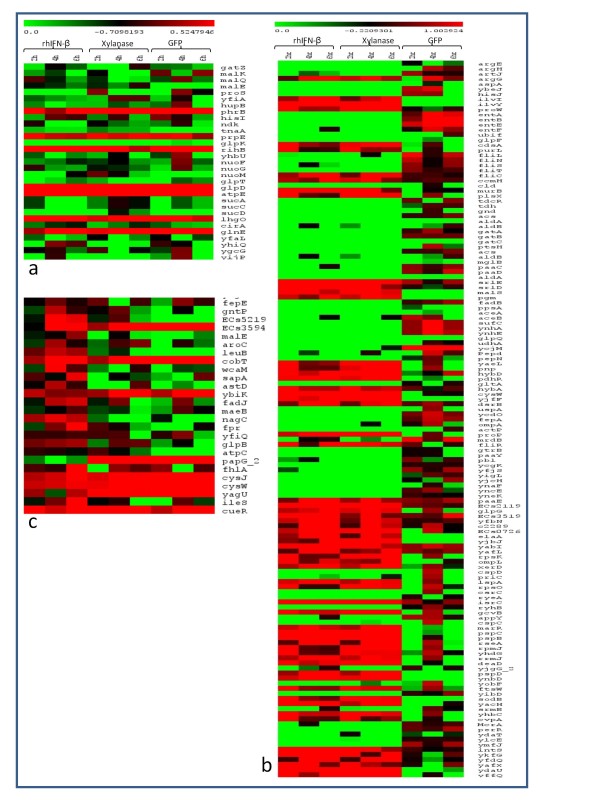
**Heat maps comparing the expression profiles**. a) Set of genes present during expression of rhIFN-β, xylanase and GFP. b) Set of genes affected during rhIFN-β and xylanase production but not in GFP. c) Set of common genes present during expression of GFP and xylanase but not in rhIFN-β.

Similarly to analyse the effect of heterologous soluble protein expression on host cells the time course expression profile of Xylanase and GFP were analysed and the genes that were solely up/down-regulated in these two sets and not in rhIFN-β (expressed as inclusion body) were picked up (Additional file 2d, g and Additional file 5) for further studies. Gene expression values of the above three sets are represented in the form of heat map in Figure 1.

## Results

**Figure 2:**
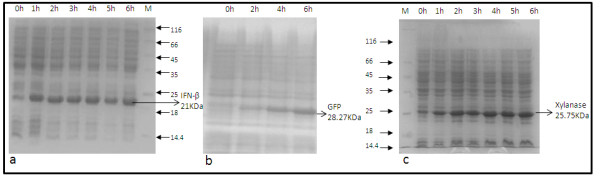
**SDS-PAGE gel picture showing total cellular protein from fed-batch culture in TB medium**. a) rhIFN-β. b) GFP c) Xylanase. Same marker lane has been used for 1(a) and 2(b).

In this work rhIFN-β was expressed as an inclusion body whereas xylanase and GFP were expressed as soluble proteins. While rhIFN-β and xylanase were expressed under a strong promoter (T7) in *E.coli *BL21 (DE3) cells, GFP was expressed under the *ara *promoter in an *E.coli *DH5α strain. Cells were grown exponentially in the bioreactor at a specific growth rate of 0.3 h^-1 ^by using an exponential feed of complex media and induction was done at an OD between 20-25. At this point the feed rate was ~40 ml/h and the OUR was 0.27 moles/l/h, with a Respiratory Quotient (RQ) of 1.1. Since the biomass yield (Y_x/s_) on glycerol, while using complex nitrogen sources had been previously determined to be between 1-1.1 g/g. The above results matched stoichiometrically and demonstrated complete consumption of substrate feed. A continuous fall in the specific growth rate was observed which dropped to zero within 4 hours of induction. In the post induction phase continuous increase in the OUR was observed which necessitated oxygen supplement of the inlet air after 1 h of induction. From the on-line metabolic activity measurement we could identify 3 phases in the metabolic activity of the culture. In the first phase from the point of induction till 2 hours the activity as measured by OUR, CER and RPM^2 ^kept increasing, even though there was continuous decline in specific growth rate. Clearly a large part of this metabolic activity was diverted towards maintenance (Russell and Cook 1995). The specific product formation rate was high during this period. Since the metabolic activity doubled in this period, the post-induction feed was also increased concomitantly (Ramalingam et al. 2007). In the second phase between 2 to 4 hours the feed was kept constant since the on-line measurement indicated a constant metabolic activity. Finally after 4 hours there was a decline in metabolic activity and the specific product formation rate declined to reach zero in 6 hours. Samples were collected to represent these three phases 2, 4 and 6 hour (post-induction). Figure 2 shows the SDS-PAGE gel picture of rhIFN-β, xylanase and GFP expression profile post induction.

### Identifying the similarities in the cellular stress response

The transcriptomic profiles of three different fermenter runs with rhIFN-β/BL21 (DE3), Xylanase/BL21 (DE3) and GFP/DH5α were analyzed post induction and genes with an expression fold change ≥2 with respect to the point of induction were chosen for further analysis. From these, the common list of genes with a high fold change across all time points and across all three fermenter runs was identified (Additional file 3). We observed that in all the three cases, the genes associated with metabolic activity in terms of carbon utilization and energy generation pathways were severely down-regulated. This was similar to earlier reports, where the expression of plasmid based proteins caused a down-regulation of genes involved in biosynthetic pathway, energy metabolism and central carbon metabolism (Ow et al. 2010).

Among the existing transport systems involved in nutrient uptake in *E.coli*, two major components of the glycerol uptake system are *glpT *(Glycerol-3-phosphate transporter) and *glpK *(Glycerol kinase). Both these were down-regulated 3.7 and 5.6 folds respectively. Oh and Liao (2000) have also reported that when glycerol was used as a carbon source, under nutrition limitation, genes involved in glycerol catabolism were down-regulated. We also observed that maltose transporters *malT, malE *and *malK *were repressed with a concomitant up-regulation of *mlc *which negatively regulates the ATP-binding component of the maltose ABC transporter (Plumbridge 2002) similar to observations of Lemuth et.al. (2008), which indicates that transport of carbon sources were significantly affected.

**Figure 3:**
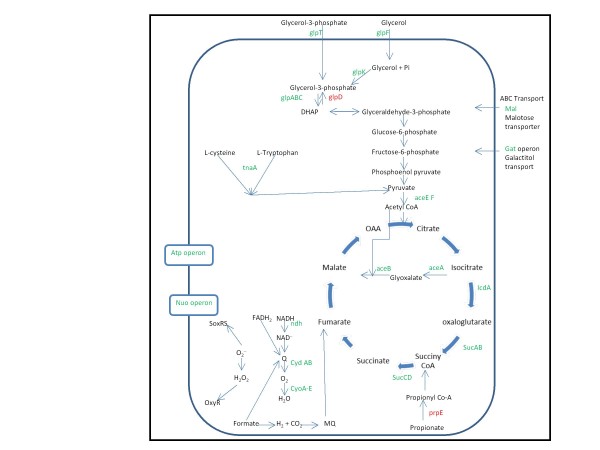
**Schematic diagram showing common genes which were up/down-regulated (fold change ≥ 2) during rhIFN-β, xylanase and GFP production, along with the processes and reactions they are involved**. Red and green colour letters represent up-regulated and down-regulated genes respectively.

The transcript levels of a number of aerobic respiration proteins involved in ATP synthesis were found to be relatively lower. The genes of the *nuo *operon encoding for components of NADH dehydrogenase-I were down-regulated. NADH: ubiquinone oxidoreductase-I (NDH-1) is an NADH dehydrogenase which is part of both the aerobic and anaerobic respiratory chain of the cell (Hua et al. 2004). It was found that the *ndh *and genes of the *atp *operon were down-regulated in line with previous observations (Durrschmid et al. 2008; Haddadin and Harcum 2005). In addition, expression of two main aerobic terminal oxidases, cytochrome bd (*cydAB*) and cytochrome bo (*cyoABCD *genes) were also reduced (Oh and Liao 2000). Concomitantly we observed a severe down-regulation of genes involved in TCA cycle (*icdA, aceBAK, acs*) and amino acid synthesis which can be attributed to the cellular stress associated with the over-expression of recombinant proteins. s*ucABCD *operon of TCA cycle was down-regulated and this may be due to the repressor activity of *ArcA/ArcB*, which is known to act on aerobic central metabolism pathway during oxidative stress (Vemuri et al. 2005). Both *glpD*, which catalyses the conversion of glycerol-3-phosphate to dihydroxyacetone phosphate, and *prpE*, a key enzyme in propionate degradation were up-regulated 10.4 fold and 5.4 fold respectively. This indicates that alternative pathways for substrate utilization are active during stress, and act as anapleurotic reactions to replenish TCA cycle metabolites. g*atZ *is involved in galactitol degradation which catalyze the dissociation of D-tagatose 1, 6-biphosphate to glycolytic intermediates (Nobelmann and Lengeler 1996). This gene was observed to be down-regulated, indicating that potential anapleurotic pathways which are energy consuming are down-regulated in order to conserve energy. Interestingly there was also down-regulation of *tnaA *which breaks down L- tryptophan and L- cysteine to pyruvate. This shows that while the overall flux in the glycolytic pathway is decreased, a cascade of events also takes place to maintain the pool of critical intermediaries inside the cell. We can therefore hypothesize that the cell ensures its supply of nodal metabolites while it reprogrammes its machinery upon induction of metabolic stress. The schematic of the processes and reactions catalyzed by this common set of differentially expressed genes is given in Figure 3.

### Analysis of differential expression due to hyper-expression

**Figure 4:**
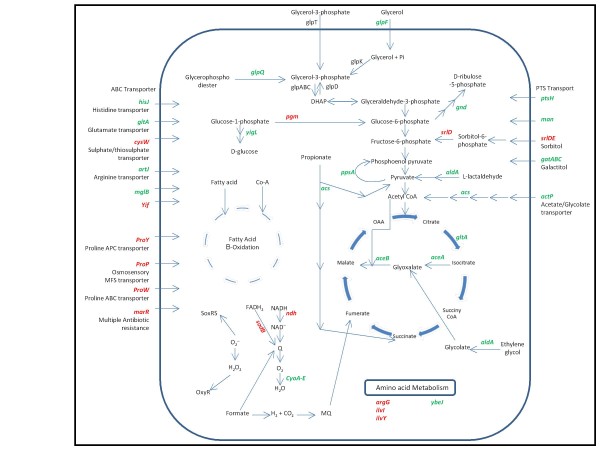
**Schematic diagram showing common genes which are up/down-regulated (fold change ≥ 2) during rhIFN-β and xylanase but not in GFP, along with the processes and reactions they are involved**. Red and green colour letters represent up-regulated and down-regulated genes respectively. (In Fig 4 and Fig 5, Black colour genes are those genes which are not present in the common gene list or does not pass the fold change cut off criteria but shown only to maintain the continuity of the steps in the important pathways)

The set of genes which were found to be up/down-regulated (fold change ≥ 2) during high level expression of rhIFN-β and xylanase under the T7 promoter, but not in the relatively lower *'ara' *based expression of GFP were analysed to understand the host response towards hyper-expression of proteins (Figure 4, Additional file 4).

The processes of cell growth and expression of foreign gene products both compete for the use of various intracellular resources for the biosynthesis, of amino acids, nucleotides as well as metabolic energy. When recombinant proteins are over-expressed under strong promoters, a major chunk of the flux of the precursors are diverted towards heterologous gene expression (Chou 2007). This gross imbalance in the resource distribution leads to degradation of cellular health and the cellular physiology is significantly reprogrammed. We thus observed that this list contained the maximum number of up/down-regulated genes. This included the major channels of precursor molecules like transporters (*artJ*, *mglB*, *hisJ*, *ybeJ, ptsH, sufC, ycdO, gatA, gatB, gatC, fepA, ompA, actP and mrdB)*, central intermediary metabolism (*pdhR*, *aceE, aceF*, *lpdA, and gltA)*, amino acid metabolism (*argE*, *argH*, *entA, entB, entE*, *entF*, *aspA *and *ubiF) *and energy generation pathways genes which were down-regulated.

*glpF*, the glycerol facilitator, which helps in facilitated diffusion of glycerol across the inner membrane of the cell was found to be down-regulated 3 fold. Down-regulation of glycerol transport and utilization pathway is a major bottleneck in achieving high yield of recombinant protein, and co expression of *glpF *with target protein has been reported to increase productivity (Choi et al. 2003). This is in agreement with the hypothesis that the cell restricts the supply of precursor molecules in order to slowdown metabolic fluxes and thus restricts foreign protein expression.

We observed that the whole *atp *operon was down-regulated, supporting the fact that energy generation pathway are repressed during metabolic stress. Simultaneously the flagellar motility (*fliL, fliN, fliS, fliT*) genes were also found to be down-regulated. A steep proton gradient is required for flagellar motility between the periplasmic space and the cytoplasm; decreased motility could indicate energy deficiency. Probably, the cell strategically also down-regulates genes related to flagellar motility to minimize energy expenditure, which is in agreement with earlier data (Jozefczuk et al. 2010). The genes *proW *and *proP *help in maintaining osmotic homeostasis, prevent cell dehydration and restore membrane turgor (Gunasekera et al. 2008; Mellies et al. 1995). These were found to be 6.0 fold and 5.3 fold up-regulated respectively, which is in agreement with the fact that hyper-expression of recombinant proteins not only affects the biosynthetic pathways but also leads to the disruption of cellular integrity. Similarly, *yaeL *was up-regulated which is activated in responses to unfolded protein stress (Alba et al. 2002; Betton et al. 1996; Jones et al. 1997; Mecsas et al. 1993; Missiakas et al. 1996). The *pnp *gene which encodes for PNPase and has a role in mRNA degradation during carbon starvation (Kaplan and Apirion 1974, 1975), was observed to be up-regulated. Interestingly these proteases and genes for mRNA degradation were not differentially expressed in case of GFP expression indicating that under lower levels of recombinant protein expression these stringent responses were not generated.

### Comparing soluble and insoluble forms of expression

**Figure 5:**
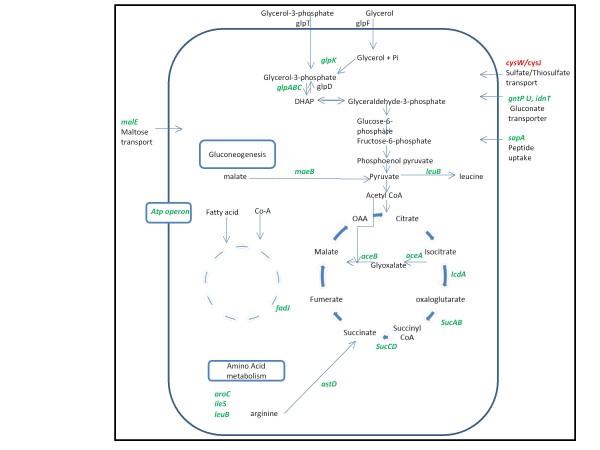
**Schematic diagram showing common genes which are up/down-regulated (fold change ≥ 2) during GFP and xylanase but not in rhIFN-β, along with the processes and reactions they are involved**. Red and green colour letters represent up-regulated and down-regulated genes respectively.

An interesting comparison of the transcriptomic profile could be made by looking at those genes which were up or down-regulated, when xylanase and GFP were expressed as soluble proteins but not during the expression of rhIFN-β (as IBs). In both cases there is a metabolic flux diversion towards product formation. However with soluble protein expression, an additional stress is imposed by the interaction of the soluble protein with the cellular constituents, which is absent when the product gets sequestered as IBs. This list of genes is given in Additional file 5 and a schematic representing the reactions and processes which are up/down-regulated are shown in Figure 5.

The amino acid biosynthetic genes, *aroC *coding for chorismate synthase, which is the key branch-point intermediate in aromatic biosynthesis, *leuB *and *ileS *were among the significantly down-regulated group. Genes involved in the anapleurotic pathways of TCA cycle intermediates *astD*, as well as the glycerol degradation genes encoded by *glpABC *operon which provides intermediaries to the glycolytic pathways were also down-regulated. The rate limiting steps of both glycolysis as well as TCA cycle were down-regulated which would result in retarded substrate utilization and energy generation pathways.

*sapA *is well known as a peptide transporter which is part of the defence degradation system in *E.coli *(Parra-Lopez et al. 1993). Along with this ATP binding to SapD has also been shown to be sufficient for restoring K^+ ^uptake in *E. coli *via its two Trk potassium transporters (Harms et al. 2001). There was a significant down-regulation of *sapA *involved in potassium uptake in *E. coli *indicating that there is a decline in nutrient uptake and oxygen consumption rate of the cell (Harms et al. 2001). Similarly the *fadJ *gene which is a part of the anaerobic β-oxidation of fatty acids was also down-regulated suggesting that the cells were not able to use fatty acids as carbon and energy source (Campbell et al. 2003). In *E. coli*, *fpr *participates in the synthesis of methionine, dissimilation of pyruvate, and synthesis of deoxyribonucleotides. The latter two reactions are anaerobic processes. In all cases, *fpr *functions together with flavodoxin in the transfer of electrons from NADPH to an acceptor (Bianchi et al. 1995; Ow et al. 2006) and this was also found to be down-regulated. *atpC *component of ATP Synthase F1 complex was down-regulated. These results indicate that the expression of a soluble protein leads to an enhanced suppression of key metabolic pathways, adversely affecting the cellular health and productivity of the host.

## Discussion

It was observed that the cellular response to the diversion of metabolites for product formation, is at multiple levels directed both at growth rate and protein production. Since growth rate and protein synthesis share common pathways, this stress response hits both processes simultaneously, affirming previous reports on the growth associated nature of recombinant protein production (Bentley et al. 1990; Shin et al. 1998). The stress response first affects the carbon uptake by down-regulating various transporters and this phenomenon was observed for all the conditions irrespective of the nature and level of recombinant protein expression. Simultaneously the carbon utilization and energy generation pathways starting from Glycolysis, TCA to electron transport chain were severely repressed resulting in decreased growth yield, product formation and viability of the cell population as has been shown by Hardiman et al. (2007).

Interestingly, there was a significant time lag between this transcriptomic down regulation and its resultant phenotype. Thus the metabolic activity which is linked to substrate uptake rate fell only after 4 hours post-induction. The down-regulation of energy generating pathways also leads to a drop in growth rate (Kasimoglu et al. 1996; Troein et al. 2007) which was also observed in the present case. It has been previously reported that in complex medium, several genes of energy generating pathways such as *hycB, cyoA, cydA, and ndh*, were down-regulated, along with the ATP synthase gene (Oh and Liao 2000), which is similar to our observations.

The addition targets of this metabolic stress response were the amino acid uptake, peptide uptake and amino acid biosynthetic pathways. Interestingly amino acid uptake and biosynthesis was significantly repressed only when soluble proteins were expressed under different promoters, whereas these pathways were not significantly affected when the recombinant protein was expressed as an inclusion body.

We observed that hyper-expression of recombinant protein tends to generate a very strong response where several pathways are affected, most importantly the transporters and the cellular degradation machinery like the osmoprotectants (*proP *and *proW*), proteases (*yaeL*) and mRNA degradation (*pnp*). All these genes were highly up-regulated during protein production with the T7 promoter (rhIFN-β and xylanase), whereas these were not significantly affected during protein production with the weaker *ara *promoter. The large fold changes in the genes associated with transport is an indication of cellular shutdown. Simultaneously the cell loses its osmotolerant property along with an increase in protease and mRNA degradation activity.

We can therefore conclude that both the nature and level of recombinant protein expression leads to the generation of a common as well as a differential stress response. Host cell engineering should take into account the nature of protein to be expressed for designing improved platforms for over-expression.

## Competing interests

The authors declare that they have no competing interests.

## Supplementary Material

Additional file 1 **Pre-induction graphs for fed-batch fermentation of GFP**. OD_600 _Vs Time. OUR(mol/l/h) Vs Time. CER(mol/l/h) Vs Time. OUR(mol/l/h) Vs RPM^2^. CER(mol/l/h) Vs RPM^2^ Click here for file

Additional file 2 **Experimental design for data analysis**. a) Set of up/down-regulated gene across different time points (2 h, 4 h and 6 h). b) Set of genes up -regulated in rhIFN-β, xylanase and GFPpe) Set of genes down-regulated in rhIFN-β, xylanase and GFP.pc) Set of genes up -regulated in rhIFN-β and xylanase but not in GFP.pf) Set of genes down-regulated in rhIFN-β and xylanase but not in GFP. d) Set of genes up -regulated in xylanase and GFP but not in rhIFN-β. g) Set of genes down-regulated in xylanase and GFP but not in rhIFN-β. Click here for file

Additional file 3 **List of common genes present during expression of rhIFN-β, xylanase and GFP with their log2 fold change values (fold change ≥ 2)**. Click here for file

Additional file 4 **List of common genes present during expression of rhIFN-β and xylanase but not in GFP, along with their log2 fold change values (fold change ≥ 2)**. Click here for file

Additional file 5 **List of common genes present during expression of GFP and xylanase but not in rhIFN-β, along with their log2 fold change values (fold change ≥ 2)**. Click here for file

## References

[B1] Acosta-Rivero N Sanchez JC Morales J Improvement of human interferon HUIFNalpha2 and HCV core protein expression levels in *Escherichia coli *but not of HUIFNalpha8 by using the tRNA(AGA/AGG) Biochemical and biophysical research communications 2002 296 5 1303 1309 10.1016/S0006-291X(02)02056-9 12207916

[B2] Alba BM Leeds JA Onufryk C Lu CZ Gross CA DegS and YaeL participate sequentially in the cleavage of RseA to activate the sigma(E)-dependent extracytoplasmic stress response Genes & development 2002 16 16 2156 2168 10.1101/gad.1008902 12183369 PMC186436

[B3] Babaeipour V Shojaosadati SA Robatjazi SM Khalilzadeh R Maghsoudi N Over-production of human interferon-[gamma] by HCDC of recombinant Escherichia coli Process Biochemistry 2007 42 1 112 117

[B4] Bentley WE Mirjalili N Andersen DC Davis RH Kompala DS Plasmid-encoded protein: the principal factor in the "metabolic burden" associated with recombinant bacteria Biotechnol Bioeng 1990 35 7 668 681 10.1002/bit.260350704 18592563

[B5] Betton JM Boscus D Missiakas D Raina S Hofnung M Probing the structural role of an alpha beta loop of maltose-binding protein by mutagenesis: heat-shock induction by loop variants of the maltose-binding protein that form periplasmic inclusion bodies Journal of molecular biology 1996 262 2 140 150 10.1006/jmbi.1996.0504 8831785

[B6] Bhattacharya P Pandey G Srivastava P Mukherjee K Combined effect of protein fusion and signal sequence greatly enhances the production of recombinant human GM-CSF in *Escherichia coli *Molecular Biotechnology 2005 30 2 103 115 10.1385/MB:30:2:103 15920280

[B7] Bianchi V Haggard-Ljungquist E Pontis E Reichard P Interruption of the ferredoxin (flavodoxin) NADP+ oxidoreductase gene of *Escherichia coli *does not affect anaerobic growth but increases sensitivity to paraquat Journal of bacteriology 1995 177 15 4528 4531 7635836 10.1128/jb.177.15.4528-4531.1995 PMC177208

[B8] Bolstad BM Irizarry RA Astrand M Speed TP A comparison of normalization methods for high density oligonucleotide array data based on variance and bias Bioinformatics 2003 19 2 185 193 10.1093/bioinformatics/19.2.185 12538238

[B9] Bowers LM Lapoint K Anthony L Pluciennik A Filutowicz M Bacterial expression system with tightly regulated gene expression and plasmid copy number Gene 2004 340 1 11 18 10.1016/j.gene.2004.06.012 15556290

[B10] Campbell JW Morgan-Kiss RM Cronan EJ A new *Escherichia coli *metabolic competency: growth on fatty acids by a novel anaerobic β-oxidation pathway Mol Microbiol 2003 47 3 793 805 10.1046/j.1365-2958.2003.03341.x 12535077

[B11] Choi JH Lee SJ Lee SY Enhanced production of insulin-like growth factor I fusion protein in *Escherichia coli *by coexpression of the down-regulated genes identified by transcriptome profiling Appl Environ Microbiol 2003 69 8 4737 4742 10.1128/AEM.69.8.4737-4742.2003 12902266 PMC169106

[B12] Chou CP Engineering cell physiology to enhance recombinant protein production in *Escherichia coli* Appl Microbiol Biotechnol 2007 76 3 521 532 10.1007/s00253-007-1039-0 17571257

[B13] Durrschmid K Reischer H Schmidt-Heck W Hrebicek T Guthke R Rizzi A Bayer K Monitoring of transcriptome and proteome profiles to investigate the cellular response of *E. coli *towards recombinant protein expression under defined chemostat conditions J Biotechnol 2008 135 1 34 44 10.1016/j.jbiotec.2008.02.013 18405993

[B14] Gill RT Valdes JJ Bentley WE A comparative study of global stress gene regulation in response to overexpression of recombinant proteins in *Escherichia coli* Metab Eng 2000 2 3 178 189 10.1006/mben.2000.0148 11056060

[B15] Gunasekera TS Csonka LN Paliy O Genome-wide transcriptional responses of *Escherichia coli *K-12 to continuous osmotic and heat stresses Journal of bacteriology 2008 190 10 3712 3720 10.1128/JB.01990-07 18359805 PMC2395010

[B16] Gupta JC Jaisani M Pandey G Mukherjee KJ Enhancing recombinant protein yields in *Escherichia coli *using the T7 system under the control of heat inducible λPL promoter J Biotechnol 1999 68 2-3 125 134 10.1016/S0168-1656(98)00193-X 10194853

[B17] Haddadin FT Harcum SW Transcriptome profiles for high-cell-density recombinant and wild-type *Escherichia coli* Biotechnol Bioeng 2005 90 2 127 153 10.1002/bit.20340 15742388

[B18] Hale RS Thompson G Codon optimization of the gene encoding a domain from human type 1 neurofibromin protein results in a threefold improvement in expression level in *Escherichia coli* Protein Expression and Purification 1998 12 2 185 188 10.1006/prep.1997.0825 9518459

[B19] Harms C Domoto Y Celik C Rahe E Stumpe S Schmid R Nakamura T Bakker EP Identification of the ABC protein SapD as the subunit that confers ATP dependence to the K+-uptake systems Trk(H) and Trk(G) from *Escherichia coli *K-12 Microbiology 2001 147 Pt 11 2991 3003 11700350 10.1099/00221287-147-11-2991

[B20] Hua Q Yang C Oshima T Mori H Shimizu K Analysis of gene expression in *Escherichia coli *in response to changes of growth-limiting nutrient in chemostat cultures Appl Environ Microbiol 2004 70 4 2354 2366 10.1128/AEM.70.4.2354-2366.2004 15066832 PMC383082

[B21] Joly JC Swartz JR In vitro and in vivo redox states of the *Escherichia coli *periplasmic oxidoreductases DsbA and DsbC Biochemistry 1997 36 33 10067 10072 10.1021/bi9707739 9254601

[B22] Jones CH Danese PN Pinkner JS Silhavy TJ Hultgren SJ The chaperone-assisted membrane release and folding pathway is sensed by two signal transduction systems The EMBO journal 1997 16 21 6394 6406 10.1093/emboj/16.21.6394 9351822 PMC1170246

[B23] Jones KL Kim SW Keasling JD Low-copy plasmids can perform as well as or better than high-copy plasmids for metabolic engineering of bacteria Metab Eng 2000 2 4 328 338 10.1006/mben.2000.0161 11120644

[B24] Jozefczuk S Klie S Catchpole G Szymanski J Cuadros-Inostroza A Steinhauser D Selbig J Willmitzer L Metabolomic and transcriptomic stress response of *Escherichia coli* Molecular systems biology 2010 6 364 20461071 10.1038/msb.2010.18 PMC2890322

[B25] Kaplan R Apirion D The involvement of ribonuclease I, ribonuclease II, and polynucleotide phosphorylase in the degradation of stable ribonucleic acid during carbon starvation in *Escherichia coli* The Journal of biological chemistry 1974 249 1 149 151 4358625

[B26] Kaplan R Apirion D Decay of ribosomal ribonucleic acid in *Escherichia coli *cells starved for various nutrients The Journal of biological chemistry 1975 250 8 3174 3178 1091648

[B27] Kasimoglu E Park SJ Malek J Tseng CP Gunsalus RP Transcriptional regulation of the proton-translocating ATPase (atpIBEFHAGDC) operon of *Escherichia coli*: control by cell growth rate Journal of bacteriology 1996 178 19 5563 5567 8824597 10.1128/jb.178.19.5563-5567.1996 PMC178391

[B28] Kiefer H Vogel R Maier K Bacterial expression of G-protein-coupled receptors: prediction of expression levels from sequence Receptors & channels 2000 7 2 109 119 10952088

[B29] Kim YS Seo JH Cha HJ Enhancement of heterologous protein expression in *Escherichia coli *by co-expression of nonspecific DNA-binding stress protein, Dps Enzyme and Microbial Technology 2003 33 4 460 465 10.1016/S0141-0229(03)00148-0

[B30] Kurland CG Dong H Bacterial growth inhibition by overproduction of protein Mol Microbiol 1996 21 1 1 4 10.1046/j.1365-2958.1996.5901313.x 8843428

[B31] Mecsas J Rouviere PE Erickson JW Donohue TJ Gross CA The activity of sigma E, an *Escherichia coli *heat-inducible sigma-factor, is modulated by expression of outer membrane proteins Genes & development 1993 7 12B 2618 2628 10.1101/gad.7.12b.2618 8276244

[B32] Meerman HJ Georgiou G Construction and characterization of a set of *E. coli *strains deficient in all known loci affecting the proteolytic stability of secreted recombinant proteins Bio/technology 1994 12 11 1107 1110 10.1038/nbt1194-1107 7765553

[B33] Mellies J Wise A Villarejo M Two different *Escherichia coli *proP promoters respond to osmotic and growth phase signals Journal of bacteriology 1995 177 1 144 151 8002611 10.1128/jb.177.1.144-151.1995 PMC176566

[B34] Missiakas D Betton JM Raina S New components of protein folding in extracytoplasmic compartments of *Escherichia coli *SurA, FkpA and Skp/OmpH Mol Microbiol 1996 21 4 871 884 10.1046/j.1365-2958.1996.561412.x 8878048

[B35] Nobelmann B Lengeler JW Molecular analysis of the gat genes from *Escherichia coli *and of their roles in galactitol transport and metabolism Journal of bacteriology 1996 178 23 6790 6795 8955298 10.1128/jb.178.23.6790-6795.1996 PMC178577

[B36] Oh MK Liao JC DNA microarray detection of metabolic responses to protein overproduction in *Escherichia coli* Metab Eng 2000 2 3 201 209 10.1006/mben.2000.0149 11056062

[B37] Ow DS-W Nissom PM Philp R Oh SK-W Yap MG-S Global transcriptional analysis of metabolic burden due to plasmid maintenance in *Escherichia coli *DH5[alpha] during batch fermentation Enzyme and Microbial Technology 2006 39 3 391 398 10.1016/j.enzmictec.2005.11.048

[B38] Ow DS Lim DY Nissom PM Camattari A Wong VV Co-expression of Skp and FkpA chaperones improves cell viability and alters the global expression of stress response genes during scFvD1.3 production Microb Cell Fact 2010 9 22 10.1186/1475-2859-9-22 20388215 PMC2868799

[B39] Parra-Lopez C Baer MT Groisman EA Molecular genetic analysis of a locus required for resistance to antimicrobial peptides in *Salmonella typhimurium* The EMBO journal 1993 12 11 4053 4062 8223423 10.1002/j.1460-2075.1993.tb06089.x PMC413698

[B40] Perez-Rueda E Collado-Vides J The repertoire of DNA-binding transcriptional regulators in *Escherichia coli K-12* Nucleic Acids Res 2000 28 8 1838 1847 10.1093/nar/28.8.1838 10734204 PMC102813

[B41] Perrenoud A Sauer U Impact of global transcriptional regulation by ArcA, ArcB, Cra, Crp, Cya, Fnr, and Mlc on glucose catabolism in *Escherichia coli* Journal of bacteriology 2005 187 9 3171 3179 10.1128/JB.187.9.3171-3179.2005 15838044 PMC1082841

[B42] Plumbridge J Regulation of gene expression in the PTS in *Escherichia coli*: the role and interactions of Mlc Current opinion in microbiology 2002 5 2 187 193 10.1016/S1369-5274(02)00296-5 11934616

[B43] Ramalingam S Gautam P Mukherjee KJ Jayaraman G Effects of post-induction feed strategies on secretory production of recombinant streptokinase in *Escherichia coli* Biochemical Engineering Journal 2007 33 1 34 41 10.1016/j.bej.2006.09.019

[B44] Ratelade J Miot MC Johnson E Betton JM Mazodier P Benaroudj N Production of recombinant proteins in the lon-deficient BL21(DE3) strain of *Escherichia coli *in the absence of the DnaK chaperone Appl Environ Microbiol 2009 75 11 3803 3807 10.1128/AEM.00255-09 19346357 PMC2687262

[B45] Russell JB Cook GM Energetics of bacterial growth: balance of anabolic and catabolic reactions Microbiological reviews 1995 59 1 48 62 7708012 10.1128/mr.59.1.48-62.1995 PMC239354

[B46] Seo JH Bailey JE Effects of recombinant plasmid content on growth properties and cloned gene product formation in *Escherichia coli* Biotechnol Bioeng 1985 27 12 1668 1674 10.1002/bit.260271207 18553628

[B47] Shin CS Hong MS Kim DY Shin HC Lee J Growth-associated synthesis of recombinant human glucagon and human growth hormone in high-cell-density cultures of *Escherichia coli* Appl Microbiol Biotechnol 1998 49 4 364 370 10.1007/s002530051183 9615476

[B48] Srivastava P Mukherjee KJ Kinetic studies of recombinant human interferon-alpha (rhIFN-[alpha]) expression in transient state continuous cultures Biochemical Engineering Journal 2005 26 1 50 58 10.1016/j.bej.2005.06.004

[B49] Suzuki M Roy R Zheng H Woychik N Inouye M Bacterial bioreactors for high yield production of recombinant protein The Journal of biological chemistry 2006 281 49 37559 37565 10.1074/jbc.M608806200 17020876

[B50] Troein C Ahren D Krogh M Peterson C Is transcriptional regulation of metabolic pathways an optimal strategy for fitness? PLoS One 2007 2 9 e855 10.1371/journal.pone.0000855 17786226 PMC1959122

[B51] Vaiphei ST Pandey G Mukherjee KJ Kinetic studies of recombinant human interferon-gamma expression in continuous cultures of *E*coli J Ind Microbiol Biotechnol 2009 36 12 1453 1458 10.1007/s10295-009-0632-x 19727876

[B52] Vemuri GN Minning TA Altman E Eiteman MA Physiological response of central metabolism in *Escherichia coli *to deletion of pyruvate oxidase and introduction of heterologous pyruvate carboxylase Biotechnol Bioeng 2005 90 1 64 76 10.1002/bit.20418 15736164

[B53] Wild J Szybalski W Copy-control tightly regulated expression vectors based on pBAC/oriV Methods in molecular biology 2004 267 155 167 15269423 10.1385/1-59259-774-2:155

