# Supplementary figures and images for: Comparative transcriptomic profile analysis of fed-batch cultures expressing different recombinant proteins in Escherichia coli
Source: AMB Express. 2011 Oct 22;1:33. doi: 10.1186/2191-0855-1-33 (PMC3214799; doi:10.1186/2191-0855-1-33)

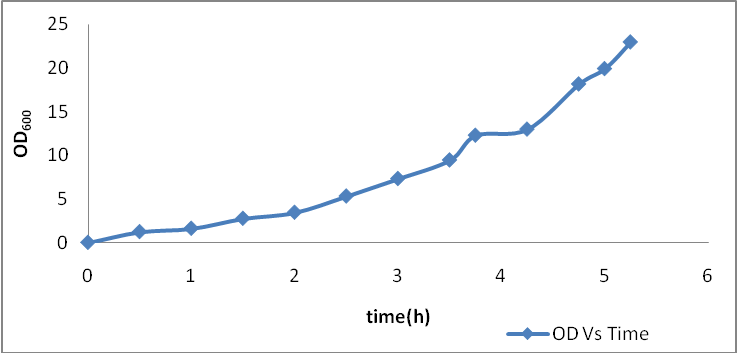


(a)


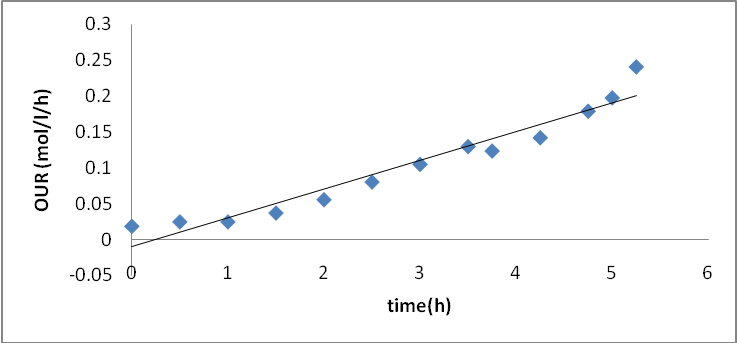


(b)


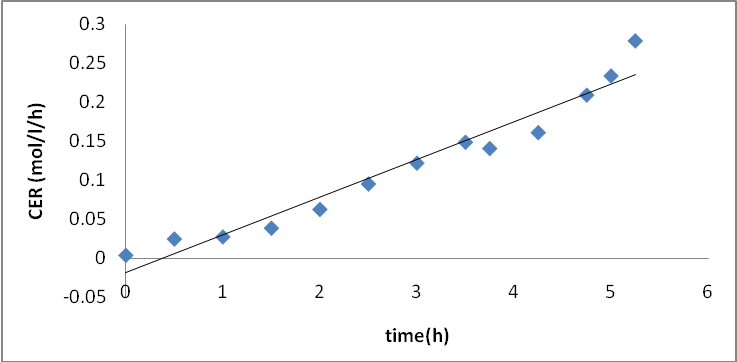


(c)


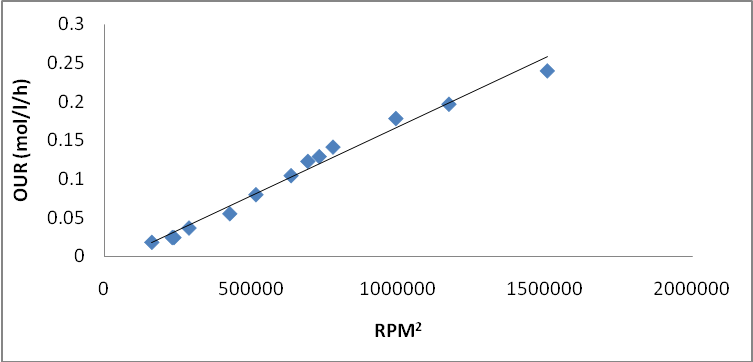


(d)


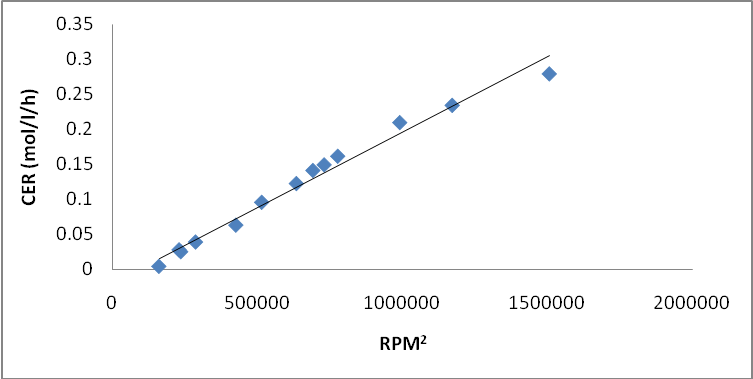


(e)

Supplement: Additional file 1 — Pre-induction graphs for fed-batch fermentation of GFP. OD600 Vs Time. OUR(mol/l/h) Vs Time. CER(mol/l/h) Vs Time. OUR(mol/l/h) Vs RPM2. CER(mol/l/h) Vs RPM2 [file 2191-0855-1-33-S1.DOC]
