# Supplementary material for: Comparative transcriptomic profile analysis of fed-batch cultures expressing different recombinant proteins in Escherichia coli
Source: AMB Express. 2011 Oct 22;1:33. doi: 10.1186/2191-0855-1-33 (PMC3214799; doi:10.1186/2191-0855-1-33)
Supplement: Additional file 2 — Experimental design for data analysis. a) Set of up/down-regulated gene across different time points (2 h, 4 h and 6 h). b) Set of genes up -regulated in rhIFN-β, xylanase and GFPpe) Set of genes down-regulated in rhIFN-β, xylanase and GFP.pc) Set of genes up -regulated in rhIFN-β and xylanase but not in GFP.pf) Set of genes down-regulated in rhIFN-β and xylanase but not in GFP. d) Set of genes up -regulated in xylanase and GFP but not in rhIFN-β. g) Set of genes down-regulated in xylanase and GFP but not in rhIFN-β. [file 2191-0855-1-33-S2.PPT]

## Slide 1
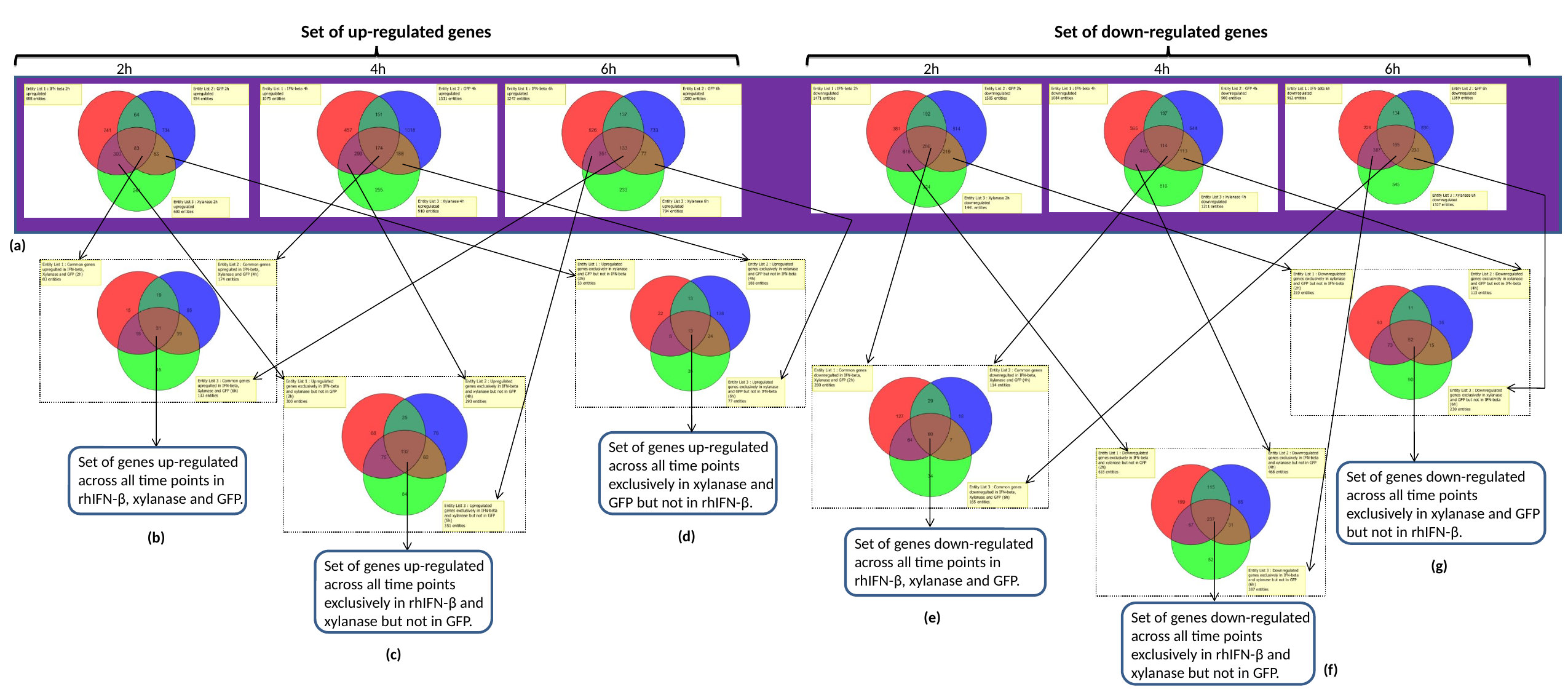

Set of down-regulated genes
Set of up-regulated genes
2h
4h
6h
2h
4h
6h
Set of genes up-regulated across all time points exclusively in xylanase and GFP but not in rhIFN-β.
Set of genes up-regulated across all time points in rhIFN-β, xylanase and GFP.
Set of genes down-regulated across all time points exclusively in xylanase and GFP but not in rhIFN-β.
Set of genes down-regulated across all time points in rhIFN-β, xylanase and GFP.
Set of genes up-regulated across all time points exclusively in rhIFN-β and xylanase but not in GFP.
Set of genes down-regulated across all time points exclusively in rhIFN-β and xylanase but not in GFP.
(d)
(b)
(g)
(e)
(c)
(f)
(a)
